# Evaluation of Commercial Self-Monitoring Devices for Clinical Purposes: Results from the Future Patient Trial, Phase I

**DOI:** 10.3390/s17010211

**Published:** 2017-01-22

**Authors:** Soren Leth, John Hansen, Olav W. Nielsen, Birthe Dinesen

**Affiliations:** 1 Laboratory of Welfare Technologies—Telehealth & Telerehabilitation, SMI, Department of Health Science and Technology, Aalborg University, Aalborg 9100, Denmark; joh@hst.aau.dk (J.H.); bid@hst.aau.dk (B.D.); 2 Medical Informatics Group, Department of Health Science and Technology, Aalborg University, Aalborg 9100, Denmark; 3 Department of Cardiology, Copenhagen University Hospital Bispebjerg, Copenhagen NV 2400, Denmark; olav.wendelboe.nielsen@regionh.dk

**Keywords:** activity tracker, pulse, physical activity, gait, slow walking, step detection, heart rate

## Abstract

Commercial self-monitoring devices are becoming increasingly popular, and over the last decade, the use of self-monitoring technology has spread widely in both consumer and medical markets. The purpose of this study was to evaluate five commercially available self-monitoring devices for further testing in clinical applications. Four activity trackers and one sleep tracker were evaluated based on step count validity and heart rate validity. Methods: The study enrolled 22 healthy volunteers in a walking test. Volunteers walked a 100 m track at 2 km/h and 3.5 km/h. Steps were measured by four activity trackers and compared to gyroscope readings. Two trackers were also tested on nine subjects by comparing pulse readings to Holter monitoring. Results: The lowest average systematic error in the walking tests was −0.2%, recorded on the Garmin Vivofit 2 at 3.5 km/h; the highest error was the Fitbit Charge HR at 2 km/h with an error margin of 26.8%. Comparisons of pulse measurements from the Fitbit Charge HR revealed a margin error of −3.42% ± 7.99% compared to the electrocardiogram. The Beddit sleep tracker measured a systematic error of −3.27% ± 4.60%. Conclusion: The measured results revealed the current functionality and limitations of the five self-tracking devices, and point towards a need for future research in this area.

## 1. Introduction

Physical inactivity and unhealthy lifestyle are major contributors to the rise in cardiovascular and chronic diseases [1]. The use of self-monitoring has proven motivational for some groups of patients with chronic disease [2]. Commercial self-monitoring devices are becoming increasingly popular, not only for consumer markets but also for research and medical purposes [3,4]. The majority of consumers use self-monitoring to optimize their fitness activities, as a means of lifestyle monitoring, and for motivational purposes. Healthcare professionals use the trackers to help patients monitor their daily activity and to motivate patients for improved physical activity. The need for physical activity in vulnerable groups such as the elderly, those with chronic diseases and obese patients has been well established. Previous studies of physical activity used self-monitoring devices to correlate body mass index and physical activity in American adults, and Izawa et al. have demonstrated correlations between mental health, mortality and physical activity in Japanese heart failure (HF) patients [5,6,7].

Modern self-monitoring technologies provide readings of step detection, sleep-depth measurements and even heart rate. Even though most self-monitoring devices are not medical devices, and are thus not approved for medical purposes, the commercially available self-monitoring devices provide low-cost, user-friendly solutions. They allow users to monitor their physical activity and daily routines, often with minimal inconvenience. Several studies have focused on the accuracy of self-monitoring devices and in a broad variety of setups [3,8,9,10,11,12,13,14,15]. Not surprisingly, the literature remains contradictory on this point, and accuracy is affected by many factors. Studies show that the step count accuracy of self-monitoring devices varies depending on height, use of walking aides, types of walking surfaces and walking speed [16,17].

Nighttime heart rate can be used as a diagnostic parameter and is associated with HF-related hospitalizations [18]. Known factors that influence the accuracy of heart rate measures in self-monitoring devices include motion artifacts caused by physical activity, skin color, and placement of trackers [19,20].

As the population of chronic disease patients increases, it is important to look for new ways of designing HF care and rehabilitation [21]. The availability and cost of activity trackers makes them ideal for use in health care systems [22]. This project is part of a three-study setup. The overall aim of the study was to identify those devices that would be most effective for HF patients in a tele-rehabilitation program as part of the Future Patient research program. The aim of this particular study was to evaluate four devices based on step count, and two devices based on heart rate for further testing in clinical applications.

## 2. Materials and Methods

This study evaluates four activity trackers, and one sleep tracker. The trackers were selected based on availability, battery life, price, previous experience in the research group and their capacity to provide access to an application interface [2]. The self-monitoring devices were evaluated in a two-step setup: First, the step count validity is evaluated through a standardized walking test. Second, the pulse accuracy is evaluated during daily living. Two armbands are tested in this setup, the Fitbit Charge HR, and the Garmin Vivofit 2. Both devices are equipped with an accelerometer and are able to estimate step count through the repetitive movements of the arms during walking. In addition, the Charge HR is also able to estimate pulse from an optical heart rate sensor placed under the armband. The Fitbit Zip and the Fitbit One are two clip-based pedometers usually worn at the hip, in a pocket or attached to the bra. Both devices are capable of measuring step count based on an accelerometer. The Beddit sleep sensor is tested for pulse accuracy. The Beddit is placed in the bed, under the sheets at chest level, and measures mechanical impulse through ballistocardiography. An overview of all devices involved in this study is shown in Table 1.

### 2.1. Standardized Walking Tests

Participants in the step validity study were 22 healthy volunteers (11 female) aged 22–52 (Mean ± SD, 31.1 ± 8.03). Participants did not suffer from any walking disabilities that could lead to unnatural walking patterns. Prior to the start of the experiment, each participant signed an informed consent form. The study was reported to the local ethics committee, although it did not require ethical approval.

**Figure 1:**
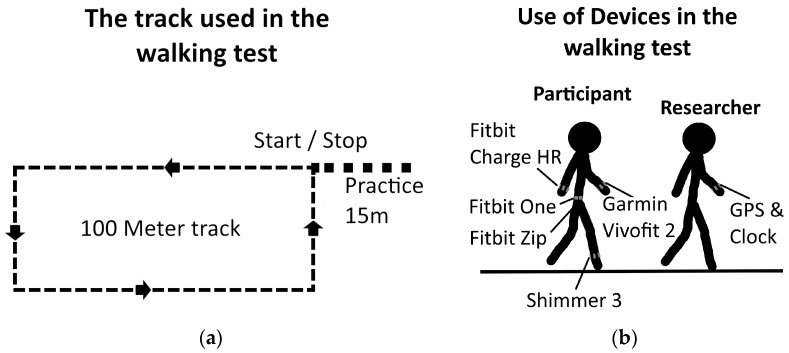
The two figures illustrate the setup used in the walking tests. (**a**) A drawing of the track used in the walking tests together with the 15 m practice line; Trackers were applied as shown in (**b**). By walking in front of the subjects, the researcher could maintain the proper pace.

During the walking tests, participants wore five devices, four activity trackers, and a Shimmer 3 used as reference. The activity trackers were set by date of birth, height, and weight, and were used according to the guidelines of the manufacturer. Participants wore the Garmin Vivofit 2 and the Fitbit Charge HR on either wrist. In order to avoid bias between dominant and non-dominant use the subjects were randomized to wear armbands on dominant and non-dominant wrist. An overview of the firmware of each device is shown in Table 1. The walking tests were performed outdoors on an asphalt parking lot. A 100-m rectangular track (6 m × 44 m) was drawn on the asphalt (Figure 1a). Each participant walked the track four times, two sessions at 2 km/h and two sessions at 3.5 km/h. The two walking speeds were selected in order to reflect the average walking speed of HF patients at 2.7 km/h [23]. The number of steps measured by each activity tracker was noted from the tracker display before and after the four walking sessions. Participants were asked to stand still during the notation and maintain a normal walking pace through the track. Before starting to walk, subjects were asked to stand at one corner of the track, where the four activity trackers were attached as described in Table 1.

A researcher controlled the walking speeds by walking in front of the participants through the walking test. Walking speeds were sustained by using a Garmin Fenix 3 GPS watch. To ensure the appropriate walking speeds, the researcher practiced his walking speed for 15 m before starting the walking test as shown on Figure 1a. The practice walking was also used to initialize the speedometer on the Garmin Fenix 3. The 15 m distance was chosen based on the time it took the Garmin Fenix 3 to provide stable measures of walking speed.

Walking at the proper pace of 2 km/h or 3.5 km/h, the researcher crossed the starting line, and the participants were asked to follow him, staying 1 m behind. The setup of the walking test is shown in Figure 1b.

The Shimmer 3 is set up to use the internal gyroscope and is attached with an elastic band to their dominant ankle. It is placed so that the z-axis of the gyroscope aligns with the transversal axis measuring the swing phase as positive rotation. The Shimmer 3 is set to sample at 64 Hz.

In addition to the GPS, a stopwatch was started when the participant crossed the starting line and stopped at the finish line. The exact average walking speeds were then calculated.

### 2.2. Calculating the Number of Steps Measured by the Shimmer 3

**Figure 2:**
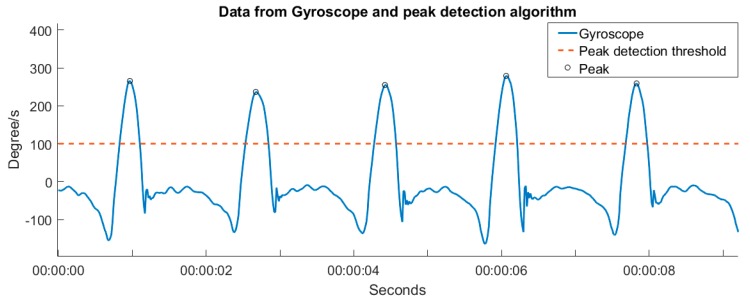
A typical sample of the Shimmer 3 data based on the walking speed of 2 km/h, and the corresponding peaks found by the algorithm.

Data from the Shimmer 3 is processed through a simple threshold algorithm. Swing phases in the gait patterns were detected by setting a 100 degree/s threshold. The number of steps was then calculated as the number of swing phases on a single leg multiplied by two. A sample of the gyroscope data and the peaks detected by the algorithm is shown in Figure 2.

To ensure that the algorithm calculated the correct number of steps, all gyroscope data was also manually inspected. Any deviation between the algorithm and the manual count was evaluated by two researchers. Eighteen deviations were found and corrected in the last step of the walking tests where the swing phase did not reach the 100 degree/s threshold.

### 2.3. Heart Rate Validity

Nine healthy participants (five females) enrolled voluntarily in the heart rate study. Participants were aged 21–42 (26 ± 5.1). No subjects reported any current or previous heart conditions, and all subjects had normal sinus rhythms. Before the start of the experiment, each participant signed an informed consent form.

At the beginning of study, participants received a Fitbit Charge HR armband, a Beddit sleep sensor and a tablet computer. The Fitbit Charge HR was attached to the wrists by the researcher, as described by the manufacturer. Participants were instructed to place the Beddit sensor in their bed and to use the tablet to switch it on when they went to bed and off when they woke up in the morning. Simultaneously with the two trackers, subjects also wore a Shimmer 3 three-point Electrocardiogram (ECG) monitor. Electrodes were placed on the right and left shoulders and upper right and left thighs and the Lead II ECG was acquired at sampling frequency 512 Hz. Subjects were instructed to use the equipment for one night.

To calculate heart rate, the R-peaks were detected in the ECG signals by implementing a Pan Tompkins like algorithm. The ECG segments were filtered with a rank 4 zero phase Chebyshev2 bandpass (1–40 Hz) filter. The filtered signals were then differentiated and squared. Finally, a moving average filter with a width of 0.2 s was applied. The pulse was calculated by peak distances in the signal.

To eliminate the errors caused by poor signal quality, the ECG signal was assessed manually. By comparing the pulse calculations with the raw ECG signal, only those segments without artifacts and with clear QRS complexes and R-peaks were selected for the analysis. All segments had a minimum duration of 1 h. The total duration of all ECG recordings was 97.46 h.

## 3. Results

### 3.1. Results from Walking Tests

**Figure 3:**
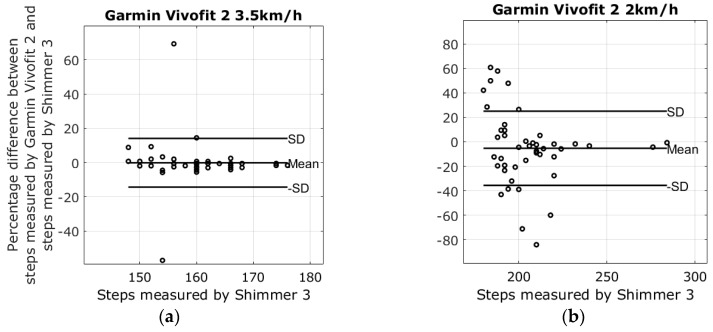
Results from the walking tests with plots showing the difference between the steps measured by the Garmin Vivofit 2 and the Shimmer 3. (**a**) The percentage error of the 3.5 km/h walking test is −0.2 ± 14.2; (**b**) The percentage error of the 2 km/h walking test −5.3 ± 30.4.

**Figure 4:**
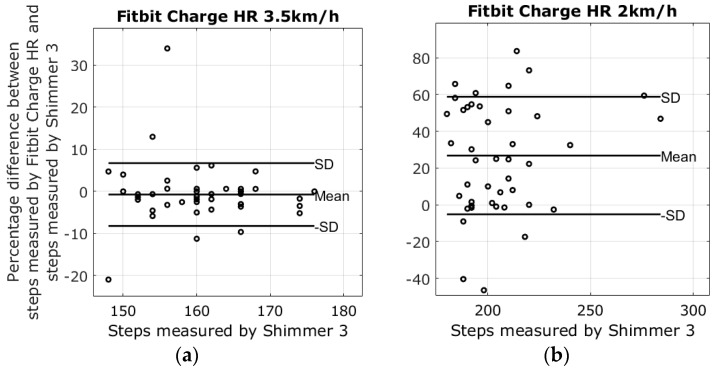
Results from the walking tests with plots showing the difference between the steps measured by the Fitbit Charge HR and the Shimmer 3. (**a**) The percentage error of the 3.5 km/h walking test is −0.7 ± 7.5; (**b**) The percentage error of the 2 km/h walking test 26.8 ± 32.0.

**Figure 5:**
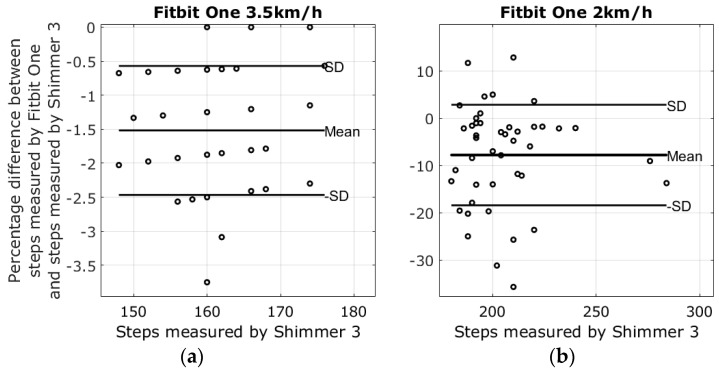
Results from the walking tests with plots showing the difference between the steps measured by the Fitbit One and the Shimmer 3. (**a**) The percentage error of the 3.5 km/h walking test is −1.5 ± 0.9; (**b**) The percentage error of the 2 km/h walking test −7.8 ± 10.5.

**Figure 6:**
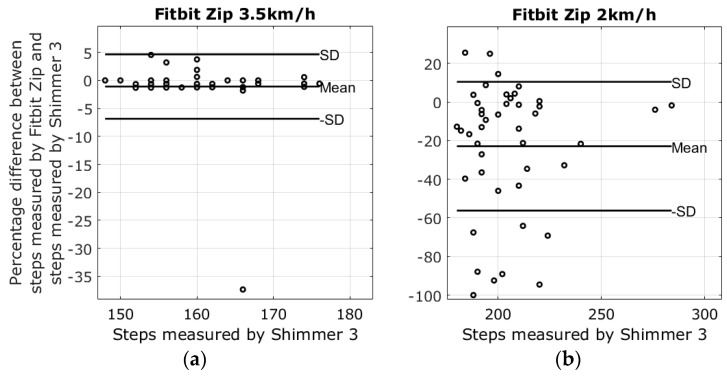
Results from the walking tests with plots showing the difference between the steps measured by the Fitbit Zip and the Shimmer 3. (**a**) The percentage error of the 3.5 km/h walking test is −1.1 ± 5.8; (**b**) The percentage error of the 2 km/h walking test −22.9 ± 33.3.

There were four different devices used to measure steps at two different walking speeds; the average number of steps in the 2 km/h walk was 205.2 ± 21.6 steps, and the average number of steps at the pace of 3.5 km/h was 160.6 ± 7.1 steps. Lap times from the stopwatch revealed that the walking speeds varied slightly between walking sessions. The mean of the 3.5 km/h walk was 3.43 ± 0.11 km/h, and the mean speed of the 2 km/h walk was 2.03 ± 0.05. Results from the walking tests are shown in the modified Bland-Altman plots in Figure 3, Figure 4, Figure 5 and Figure 6. The ordinate axis shows the percentage error as compared to the Shimmer 3 readings, and the horizontal axis show the steps measured by the Shimmer 3. Equation (1) is used to calculate the percentage error for each device.

(1) $$Step\_Error\%=\frac{Device\_Steps-Shimmer\_Steps}{Shimmer\_Steps}\times 100$$

The highest average systematic error found was with the Fitbit Charge HR at 26.8% steps too many in the 2 km/h walking test, and the lowest average systematic error was found with the Garmin Vivofit 2 at 3.5 km/h, measuring −0.2% steps too few as compared to the Shimmer 3. An overview of the results from the walking tests is shown in Table 2.

### 3.2. Results from Heart Rate Study

Heart rate measures from the ECG were rounded to the nearest integer and compared to the pulse estimates from the Fitbit Charge and the Beddit sleep monitor. To match the smoothed readings by the Beddit sleep tracker and Fitbit Charge HR, averaging windows were applied to the ECG heart rate calculations. Comparisons between the pulse measured by the Fitbit Charge HR and the heart rate measured by ECG were carried out by averaging the ECG heart rate calculations 1 min prior to each Fitbit measurement. The 1 min window was chosen based on the frequency of the Fitbit Charge HR pulse measurements. Similarly, the pulse from the Beddit was compared to the average pulse measured by the ECG during the previous 5 min. Errors were calculated based on differences between the averaged ECG heart rate calculations and the pulse estimates from the Fitbit and Beddit. The Fitbit and Beddit errors were calculated using Equations (2) and (3). Lastly, a comparison between the Fitbit and Beddit was carried out using Equation (4).

(2) $$Fitbit\_Error\%=\frac{Pulse\_Fitbit-1\min\_Average\_Pulse\_ECG}{1\min\_Average\_Pulse\_ECG}\times 100$$

(3) $$Beddit\_Error\%=\frac{Pulse\_Beddit-5\min\_Average\_Pulse\_ECG}{5\min\_Average\_Pulse\_ECG}\times 100$$

(4) $$Fitbit\_Vs\_Beddit\_Error\%=\frac{Pulse\_Fitbit-Pulse\_Beddit}{Pulse\_Beddit}\times 100$$

**Figure 7:**
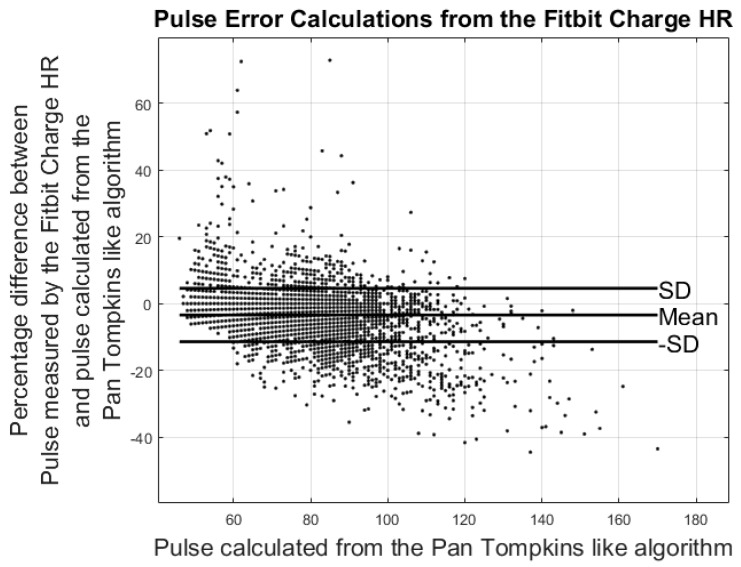
Results from the heart rate study: the plot shows percentage differences in the average pulse calculated from the Fitbit Charge HR and the Pan Tompkins-like algorithm.

**Figure 8:**
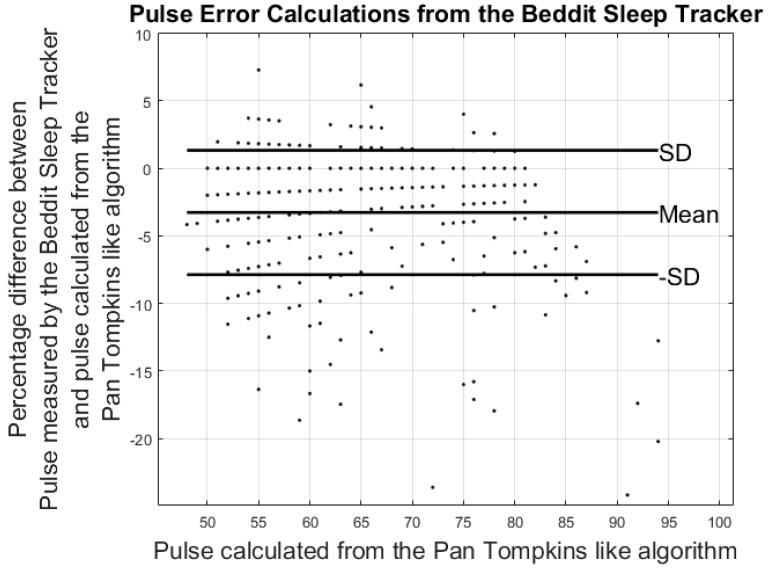
Results from the heart rate study: the plot shows differences in the average pulse calculated from the Beddit sleep tracker and the Pan Tompkins-like algorithm.

Results from the pulse error calculations are shown in the modified Bland-Altman plots in Figure 7 and Figure 8. The ordinate axis shows the percentage error as compared to the results from the Pan Tompkins algorithm and the horizontal axis shows the pulse calculated from the Pan Tompkins algorithm. Equations (2) and (3) were used to calculate the percentage error for each device. Figure 8 shows the comparison of the pulse measurements between the Fitbit Charge HR and the Beddit sleep tracker.

**Figure 9:**
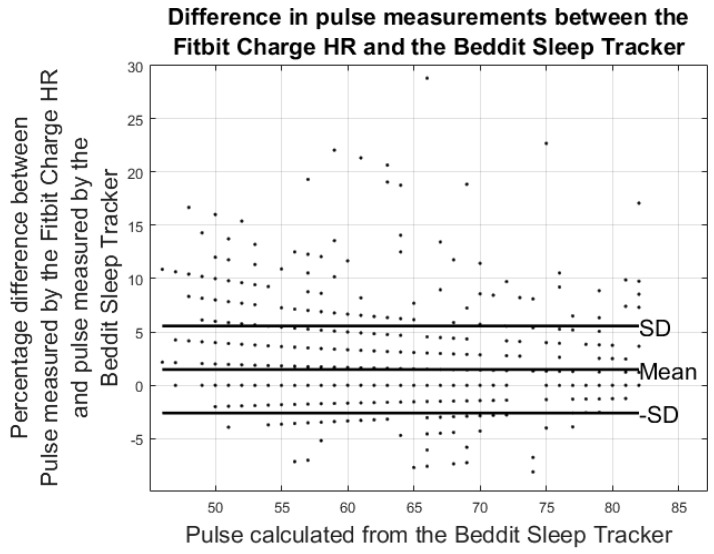
Results from the heart rate study: the plot shows percentage differences in the pulse measured by the Fitbit Charge HR and the Beddit sleep tracker.

Results from the modified Bland-Altman plots in Figure 7, Figure 8 and Figure 9 are summarized in Table 3.

## 4. Discussion

The aim of this study was to validate devices on step count and heart rate using healthy subjects. The overall aim was to identify the most sustainable devices for HF patients in a tele-rehabilitation program.

All activity trackers increased percentage errors during the 2 km/h walking test as compared to the 3.5 km/h walking test. Both the average systematic error and standard deviations were affected by the reduction in walking speed, where the Fitbit Charge, the Fitbit Zip, and the Garmin Vivofit 2 all showed standard deviations above 30.4% during the 2 km/h walking test, making their usability extremely limited in this scenario. Although gait patterns of healthy volunteers may differ from the gait of the elderly or chronically ill groups, the same patterns of negative correlations between gait speed and step accuracy were found in this study [10]. The gait speeds used in this study were selected on the basis of the average gait speeds of HF patients. However, the two gait speeds of 2 km/h and 3.5 km/h did not represent all HF patients. Based on results from Pulignano et al., the slowest group of HF patients walked an average of 1.8 km/h. Moreover, results from previous studies suggest that the step detection accuracies of self-monitoring devices at extremely slow gait <1.8 km/h are reduced substantially. Hence, self-monitoring devices may not be the most appropriate option for monitoring physical activity in extremely slow-walking groups [20,21].

The reliability of the Fitbit One found in this study corresponds with the results reported in Simpson et al. [24]. Simpson et al. tested the Fitbit One with elderly subjects at gait speeds ranging from 1.08 km/h to 3.24 km/h. Simpson et al. also reported that a waist-worn Fitbit One may be unable to detect speeds slower than 1.8 km/h. The Fitbit Zip had shown an error of −1.1% ± 5.8% steps during the 3.5 km/h walking test. However, the plot in Figure 6a shows that the results were affected by outliers; nonetheless, the result is similar to the findings of Ferguson et al. and Kooiman et al., who found that the Fitbit Zip was highly valid for measuring step count in a laboratory setting at a 4.8 km/h gait speed and free living conditions [25,26]. Evaluating the Fitbit Zip during the 2 km/h walking test increased the error to −22.9% ± 33.3%, a result similar to the treadmill study by Femina et al., who found an error of −17.27% compared to the expected steps [27]. No previous study was found evaluating the step count precision of the Fitbit Charge HR and the Garmin Vivofit 2 during slow walking speeds. The plots in Figure 3a and Figure 4a reveal that the results from the Fitbit Charge HR and the Garmin Vivofit 2 were affected by outliers during the 3.5 km/h walking test. These outliers contribute to an increase in the standard deviations and may explain the relatively high standard deviations as compared to the Fitbit One. To our knowledge, no previous study has addressed the issue of outliers in step detection for any of the self-monitoring devices evaluated in this study.

The modified Bland-Altman plots of the pulse measurements revealed an underestimation of the Fitbit Charge HR compared to the ECG measurements. These findings are supported by the work of Jo et al., who continue to conclude that especially high pulse measurements are underestimated by the Fitbit Charge HR [28]. No previous study was found evaluating the heart rate precision of the Beddit sleep sensor.

The number of participants in the pulse accuracy calculations was not large enough to fully generalize the devices’ capabilities for measuring pulse. Further studies are needed to fully validate the pulse accuracies of these devices, taking into account both the physical activity and skin color of participants. Results from the pulse measurements of the Beddit sleep tracker and the Fitbit Charge HR are not directly comparable, as the Fitbit Charge HR measures all-day pulse, while the Beddit measures pulse only during nighttime. As a result, the accuracy measures cannot be used to compare the two devices. However, the results can be used to help health care professionals become aware of the accuracies of the various self-monitoring devices and of the pulse accuracies before considering self-tracking devices for monitoring in a clinical context.

To ensure accurate heart rate calculations, the ECG was segmented. This meant that the noisier parts of the ECGs were discarded. It cannot be ruled out that this method may have favored calm periods of the ECG readings, when the participants were at rest. It follows that the comparisons of the Fitbit Charge HR and the ECG may be slightly biased towards the resting periods of the participants.

One issue that arises from the use of these devices in a clinical context is that the actual algorithms behind the heart rate estimate are unavailable. The window size picked for averaging the ECG pulse recordings was selected based on the update frequency of the pulse readings in the application interface. This method may not be the same method as that used by Fitbit and Beddit.

This study presents evaluations based only on step count and heart rate validity. However, self-monitoring devices are often capable of detecting additional variables, such as energy expenditure, distance walked and sleep depth. Evaluating the measurements of self-monitoring devices is important for their future use in clinical applications. However, a full evaluation of patient perceptions and ease of use is needed in order to assess the usability of self-tracking devices in a clinical context [29]. Further evaluations of the self-monitoring devices will be undertaken as part of the Future Patient trial (www.labwelfaretech.com) in order to fully validate them for use in chronically ill HF patients.

## 5. Conclusions

This study shows that some self-monitoring devices are better suited than others for measuring step count at slow walking speeds. No device showed an absolute systematic error above 1.5% during the 3.5 km/h gait speed. However, the standard deviations were highest in the two wrist-worn devices. During the 2 km/h gait speed, the Fitbit One and the Garmin Vivofit 2 showed the lowest average systematic error percentage; however, the standard deviations of the Fitbit One were significantly lower, making this the most reliable candidate for use in slow-walking populations.

When averaging the ECG pulse within 1 min and 5 min windows, the error percentages of pulse estimates found by the Fitbit Charge HR were −3.42% ± 7.99% and those of the Beddit sleep tracker were −3.27% ± 4.60%. The findings reveal the current functionality and limitations of commercially available self-tracking devices, and point towards a need for future research in this area.

## Figures and Tables

**Table 1:** Self-monitoring devices tested in this study.

	Garmin Vivofit 2	Fitbit Charge HR	Fitbit One	Fitbit Zip	Beddit Sleep Tracker
Firmware	3.30	122	60	86	2.0.1(103)
Device validated on step detection	✔	✔	✔	✔	
Devices validated on pulse		✔			✔
Placement	Wrist	Wrist	Hip	Hip	In Bed

**Table 2:** Results from the walking tests shown as percentage difference between the Shimmer 3 and the self-monitoring devices. Values are shown as mean ± SD.

	Garmin Vivofit 2	Fitbit Charge HR	Fitbit One	Fitbit Zip
2 km walking speed	−5.3% ± 30.4%	26.8% ± 32.0%	−7.8% ± 10.5%	−22.9% ± 33.3%
3.5 km walking speed	−0.2% ± 14.2%	−0.7% ± 7.5%	−1.5% ± 0.9%	−1.1% ± 5.8%

**Table 3:** Results from the heart rate study shown as percentage differences between the Fitbit Charge HR, the Beddit sleep tracker, and the averaged heart rate calculated from the ECG signal. Errors are shown as mean ± SD.

	Fitbit Charge HR vs. ECG-Pulse	Beddit vs. ECG-Pulse	Fitbit Charge HR vs. Beddit (Nighttime)
Pulse Error%:	−3.42% ± 7.99%	−3.27% ± 4.60%	1.49% ± 4.08%
Hours of data analyzed	88 h	36 h	42 h
